# Solitons in ultrafast semiconductor lasers with saturable absorber

**DOI:** 10.1515/nanoph-2025-0057

**Published:** 2025-08-28

**Authors:** Luigi Lugiato, Franco Prati, Massimo Brambilla, Lorenzo Luigi Columbo

**Affiliations:** Dipartimento di Scienza e Alta Tecnologia, Università dell’Insubria, Via Valleggio 11, 22100 Como, Italy; Dipartimento di Elettronica e Telecomunicazioni, 19032 Politecnico di Torino, Corso Duca degli Abruzzi 24, 10129 Torino, Italy; Como Lake Institute of Photonics, Via Valleggio 11, 22100 Como, Italy; Dipartimento di Fisica Interateneo and CNR-IFN, Università e Politecnico di Bari, Campus, Via Orabona 4, 70125 Bari, Italy

**Keywords:** solitons, quantum cascade lasers, mode locking

## Abstract

We describe structure localization and dissipative solitons in a semiconductor laser with a saturable absorber exhibiting gain/absorption recovery times shorter than the photon lifetime. Under assumptions compatible with QCL characteristics and graphene-based absorbers, we study the existence and stability of solitons, along with their dynamical behavior. Numerical simulations confirm the robustness of our predictions. This evidence hints at promising pathways to realize passive mode locking in ultrafast lasers, implying highly valuable application prospects.

## 1 Introduction

The generation of solitons in nonlinear optical systems in coherently driven resonators is strongly favored by the presence of bistability, both in the passive configuration without population inversion [1] and in the case of lasers [2]. The laser with saturable absorber (LSA, see, e.g., [3], [4] and [5] for earlier studies) is another system that can display a bistable scenario even in the absence of an external laser beam injected into the cavity, so it turns out to be much more compact than the systems considered in [1] and [2]. Moreover, it is important to note that in the LSA, the bistability occurs between a lasing state and a nonlasing state so that the soliton pedestal lies at zero intensity (i.e., at the lower branch of the hysteresis cycle), whereas in both passive systems and in lasers, it corresponds to a finite intensity continuous wave (CW) solution). Therefore, the case of LSA is favorable for the intensity contrast enhancement of the soliton and for the absence of a largely dominant central mode, which is one drawback of the frequency combs generated in driven systems. The optical frequency comb associated with the soliton in this case exploits the whole emitted field power (e.g., increasing the power per mode figure, useful for applications).

In an LSA with fast gain and absorption, the material variables adiabatically follow the electric field, whose dynamics is described by a single equation, often called the master equation. Different kinds of such equations have been widely studied in the past looking for localized solutions, which were called dissipative solitons. If the Laplacian term of the master equation describes diffraction, we speak of spatial solitons, if instead the Laplacian term is associated with the group velocity dispersion, we speak of temporal solitons. The interplay among cavity dissipation, energy intake from laser bias and nonlinearity, compensates the spreading action of diffraction and dispersion in each case.

Despite the different physical origin, the mathematical description of dissipative solitons is the same; the only important difference is that for temporal solitons, the imaginary coefficient of the Laplacian term can be positive or negative depending on whether the dispersion is anomalous or normal, while it is always positive for spatial solitons.

Spatial solitons in LSA have been extensively discussed in a sequence of theoretical articles by Rosanov and collaborators (see [6] and references therein). In those papers, the saturation of both gain and absorption is fully taken into account without approximations and, with few exceptions [7], the materials are assumed to be resonant with the electric field. This implies that the upper branch of the bistable curve is modulationally stable, unlike in passive and active systems with injection.

If the field intensity is much smaller than the saturation intensity of both the active and the passive materials, the saturation terms can be expanded into power series with respect to the field intensity. Depending on whether the expansion is arrested to the first or to the second order, the resulting equation is a Ginzburg–Landau equation with cubic or quintic nonlinearity. The cubic Ginzburg–Landau equation (CGLE), also known as Haus master equation in this context [8], can be regarded as a dissipative version of the nonlinear Schrödinger equation. As such, it admits solitonic solutions with a sech profile, which, however, are generally unstable because they do not coexist with a stable zero background. The cubic–quintic Ginzburg–Landau equation (CQGLE) overcomes this problem because it allows for bistability. Its solitonic solutions are more complex than the sech, but they are stable for certain values of the parameters [9]. For this reason, the CQGLE has become the paradigmatic model for the study of dissipative temporal solitons in an LSA [10].

In this paper, we propose a model suitable for an LSA with fast gain and absorption, a small saturation intensity for the absorber and a large saturation intensity of the active medium, much larger than that of the absorber. This model suitably describes a QCL device and turns out to be intermediate between that of Rosanov and collaborators and the CQGLE.

We assume that the field intensity is small compared to the saturation intensity of the active material but it is comparable to the saturation intensity of the passive material. Therefore, we apply a cubic approximation for the gain and keep the full saturation for absorption. This offers two main advantages: first, the cubic nonlinearity of the gain implies that the solitonic solution is well approximated by a sech, which allows an insightful, analytic description of soliton properties as in the CGLE; second, retaining the full saturation for the absorption stabilizes the solitons over a wide range of parameters.

Our aim here is to identify a new paradigmatic model for the LSA, similarly to what happened with the model of [11] for the case of a passive system and with the model of [12] for the laser case.

We start from a model formulated in [13], modified in order to describe temporal rather than spatial solitons. We adiabatically eliminate the population variables, assuming that the recovery times of both the amplifier and the absorber are much shorter than the cavity roundtrip time and, therefore, shorter than the photon lifetime. This condition is known to be favorable for the formation of solitons along the propagation direction (temporal solitons) in a macroscopic LSA configuration consisting in a Vertical Cavity Surface Emitting Laser (VCSEL) coupled to a distant, resonant, semiconductor-based, saturable absorber mirror [14]. In that case, the condition was achieved, in spite of the relatively large relaxation times of the amplifier and the absorber, by considering a very long external cavity.

On the other hand, this is the typical case of unipolar semiconductor lasers as Mid-IR or THz QCLs (or NIR semiconductor lasers based on quantum dots), where the carrier decay rate is associated with phonon scattering and thus to a characteristic time scale that typically ranges from 1 ps to 10 ps [15], [16]. For instance in [17], [18], where a mode-locked THz quantum cascade laser was very recently obtained using graphene as absorber, the reported values for the recovery times are in the range 2–3 ps for the amplifier and for the absorber, to be compared to a cavity roundtrip time of 72 ps for a 3 mm-long Fabry–Perot cavity.

In this case, evidence for soliton formation in these classes of devices would certainly have a relevant impact on applications, given the shortage of compact short pulse emitters. This situation is caused by the well-known requirement of a gain recovery time larger than the cavity round-trip time, in order to reach passive mode-locking regimes [19]. In principle, as will be clarified in the following, the shorter the gain/absorption recovery time, the better our model will represent a valid approximation of the system dynamics.

We note that, with respect to the model used to describe passive mode locking in THZ QCL with graphene saturable absorber [18], our approach leads to a single master equation for the LSA. This allows us to predict the existence of a region where ultrashort pulses form and to derive their approximated analytical expression. This clearly shows their important connection with solitons observed in other nonlinear dissipative system, similarly described by a field master equation. Hence, we believe that the results reported in this manuscript can have both an applicative and fundamental impact.

Moreover, at variance with what was proposed in [18], we show that mode-locked pulses can be stabilized not only by adding Spatial Hole Burning (SHB) terms to the Haus equation but also by simply assuming a saturation intensity in the absorber much smaller than that in the laser material.

In Section 2, we formulate the model analyzed in this article and we report on its CW and soliton solutions. In Section 3, we analyze numerically the stability of stationary and traveling solitons. Section 4 is devoted to the conclusions.

## 2 The model

We start from Eqs. (1) of [13] modified in order to account for the dynamics along the longitudinal direction *z* rather than the transverse ones and with the diffraction term replaced by a term associated with group velocity dispersion [2]

(1) $$\frac{\partial F}{\partial t}+\tilde{c}\frac{\partial F}{\partial z}=\kappa \left[\left(1-i\alpha \right)D-\left(1-i\beta \right)\bar{D}-1+id\frac{\partial^{2}}{\partial z^{2}}\right]F,$$

(2) $$\frac{\partial D}{\partial t}=\gamma \left[A-\left(1+|F{|}^{2}\right)D\right],$$

(3) $$\frac{\partial \bar{D}}{\partial t}=\bar{\gamma }\left[\bar{A}-(1+s|F{|}^{2})\bar{D}\right].$$

*F* is the normalized slowly varying envelope of the electric field, *D* and $\bar{D}$ are related to the carrier densities in the active and in the passive materials. *κ*, *γ*, and $\bar{\gamma }$ are the damping rates of the electric field, gain, and absorption, respectively. The dispersion coefficient is defined as $d=\left(-{\tilde{c}}^{3}k^{\prime \prime }\right)/\left(2\kappa \right)$ [2] where $\tilde{c}$ is the light velocity in the cavity and *k*″ is the second order dispersion coefficient of the background (waveguide). We assume anomalous dispersion, so that *d* > 0, and thus the following results rigorously apply to Mid-IR QCLs. However, we observe that although uncompensated THz QCLs [17] feature normal dispersion, recently evidence was reported on suitable techniques allowing the management of dispersion toward the anomalous regime [20].

*A* is the pump parameter of the active material and $\bar{A}$ is the absorption parameter of the passive material. The parameter *α* (*β*) is the linewidth enhancement factor [21] of the active (passive) material. Such terms account for the asymmetry of gain/absorption and dispersion curves with respect to the gain peak and are evaluated at the gain peak [22]. Finally, *s* is the ratio of the saturation intensity in the active material to the saturation intensity in the passive material.

The absorber is assumed to be homogeneously distributed along the cavity. The coefficient of the second order derivative term is purely imaginary since we assume that the waveguide dispersion dominates material contributions, which could be derived following the same procedure as in [2].

We assume $\gamma ,\bar{\gamma }\gg \kappa$ for ultrafast lasers as Mid-IR or THz QCLs and adiabatically eliminate the population variables. We also implement the field description in a comoving reference frame introducing $\bar{z}=z-\tilde{c}t$ so that the model now reads

(4) $$\frac{\partial F}{\partial t}=\kappa \left[\frac{\left(1-i\alpha \right)A}{1+|F{|}^{2}}-\frac{\left(1-i\beta \right)\bar{A}}{1+s|F{|}^{2}}-1+id\frac{\partial^{2}}{\partial {\bar{z}}^{2}}\right]F.$$

The homogeneous $\left(\partial F/\partial \bar{z}=0\right)$ and stationary (*∂F*/*∂t* = 0) solution has the form $F=\sqrt{X}{\text{e}}^{-\text{i}\omega t}$ . The oscillation frequency *ω* is given by

(5) $$\omega =\kappa \left[\alpha +\frac{\left(\alpha -\beta \right)\bar{A}}{1+sX}\right].$$

and the stationary intensity *X* is the solution of the equation

(6) $$A=\left(1+X\right)\left(1+\frac{\bar{A}}{1+sX}\right).$$

If $\bar{A}\ll 1$ (small absorption) and *X* ≪ 1 (laser close to threshold), Eq. (6) can be approximated as

(7) $$A=1+X+\frac{\bar{A}}{1+sX},$$

which implies *A* − 1 ≪ 1. Since *X* ≪ 1, the saturation term is not negligible only if *s* ≫ 1, so that *sX* is of order unity. This assumption was recently reasonably met in a THz QCL laser with multilayer graphene stripes embedded alongside the top contact acting as a distributed saturable absorber [17], [18].

**Figure 1:**
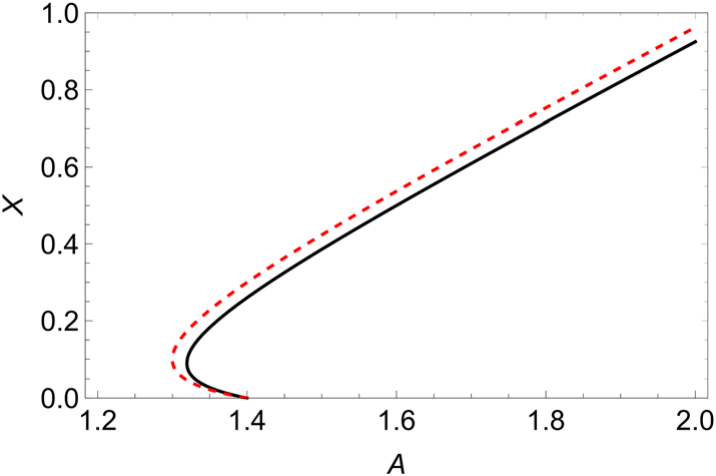
Stationary intensity as a function of the pump parameter *A* for $\bar{A}=0.4$ and *s* = 10. The black solid line is the solution of the exact Eq. (6), and the red dashed line is the solution of the approximated Eq. (7).

Figure 1 compares the solution of Eqs. (6) and (7) and shows that the agreement is very good for the chosen parameters.

According to Eq. (7), the function *A*(*X*) has a minimum in

(8) $$X_{\min}=\frac{\sqrt{s\bar{A}}-1}{s},\;A_{\min}=1+2X_{\min}+\frac{1}{s}.$$

*X*_min_ is a positive quantity if and only if

(9) $$s>\frac{1}{\bar{A}},$$

in agreement with the assumption *s* ≫ 1. If inequality (9) holds, the stationary curve *X* = *X*(*A*) has a *C* shape and the laser bifurcation is subcritical.

All these considerations suggest to introduce the scaling

$$A-1=a\epsilon ,\;\bar{A}=\bar{a}\epsilon ,\;s=\bar{s}/\epsilon ,\;F=x\sqrt{\epsilon },$$

with *ϵ* ≪ 1 and $a,\bar{a},\bar{s},x$ of order unity. Eq. (4) becomes

(10) $$\frac{\partial x}{\partial t}=\kappa \left[\left(1-i\alpha \right)\frac{\left(1+a\epsilon \right)}{1+|x{|}^{2}\epsilon }-\frac{\left(1-i\beta \right)\bar{a}\epsilon }{1+\bar{s}|x{|}^{2}}-1+id\frac{\partial^{2}}{\partial {\bar{z}}^{2}}\right]x,$$

which, neglecting terms of order *ϵ* ^2^, can be approximated as

(11) $$\begin{array}{ll} \frac{\partial x}{\partial t} & =\kappa \left[-i\alpha +\left(1-i\alpha \right)\left(a-|x{|}^{2}\right)\epsilon -\frac{\left(1-i\beta \right)\bar{a}}{1+\bar{s}|x{|}^{2}}\epsilon \right.\\ & \;\left.+id\frac{\partial^{2}}{\partial z^{2}}\right]x. \end{array}$$

The oscillating term −*iαx* can be eliminated by shifting the reference frequency by *κα*

(12) $$\frac{\partial x}{\partial t}=\kappa \left[\left(1-i\alpha \right)\left(a-|x{|}^{2}\right)\epsilon -\frac{\left(1-i\beta \right)\bar{a}}{1+\bar{s}|x{|}^{2}}\epsilon +id\frac{\partial^{2}}{\partial {\bar{z}}^{2}}\right]x.$$

Finally, to remove the smallness parameter *ϵ*, we introduce the new scaling on the time and space variables *τ* = *κϵt* and $\eta =\bar{z}\sqrt{\epsilon /d}$ and obtain

(13) $$\frac{\partial x}{\partial \tau }=\left(1-i\alpha \right)\left(a-|x{|}^{2}\right)x-\frac{\left(1-i\beta \right)\bar{a}}{1+\bar{s}|x{|}^{2}}x+i\frac{\partial^{2}x}{\partial \eta^{2}}.$$

We denote by *L* the cavity length in the dimensionless variable *η*. We substitute $\bar{a}\tau \to \tau$ , $\sqrt{\bar{a}}\eta \to \eta$ , and $x/\sqrt{\bar{a}}\to x$ and define the parameters

(14) $$\mu =\frac{a}{\bar{a}}=\frac{A-1}{\bar{A}},\;S=\bar{s}\bar{a}=s\bar{A}.$$

We then remove the parameter $\bar{a}$ to further simplify the notation and achieve the final model equation

(15) $$\frac{\partial x}{\partial \tau }=\left(1-i\alpha \right)\left(\mu -|x{|}^{2}\right)x-\frac{1-i\beta }{1+S|x{|}^{2}}x+i\frac{\partial^{2}x}{\partial \eta^{2}}.$$

The final longitudinal variable *η* turns out to be scaled to the length $\sqrt{d/\bar{A}}=\sqrt{-{\tilde{c}}^{3}k^{\prime \prime }/2\kappa \bar{A}}$ . Assuming *k*″ ∼ 10^−23^ s^2^/m, *κ* ∼ 10^10^ s^−1^, and $\bar{A}=0.4$ , this length is of the order of 50 μm.

### 2.1 The CW solution

The CW solution is the homogeneous stationary solution $x=\sqrt{X}{\text{e}}^{-\text{i}\omega \tau }$ of Eq. (15), with

(16) $$\omega =\frac{\alpha -\beta }{1+SX},\;\mu =X+\frac{1}{1+SX}.$$

The threshold now is *μ* = 1, and the bifurcation at threshold is subcritical if

(17) S>1.

In that case, the coordinates of the turning point are

(18) $$X_{\min}=\frac{\sqrt{S}-1}{S},\;\mu_{\min}=\frac{2\sqrt{S}-1}{S}.$$

The stability of the CW solution can be analytically assessed by studying its stability with respect to the growth of longitudinal modes with wave-vector *K*. The negative slope branch is always unstable as usual, even for *K* = 0. The stability of the upper branch depends on *α* and *β*. If *α* = 0, the upper branch is always stable independently of *β*. If *β* = 0 and *α* > 0, the upper branch is always unstable. If *α* ≠ 0 and *β* ≠ 0, the upper branch is unstable for *X* > *X*_*c*_ and *μ* > *μ*_*c*_, where

(19) $$X_{c}=\frac{\sqrt{S}\sqrt{\frac{\beta }{\alpha }}-1}{S},\;\mu_{c}=\frac{\sqrt{S}\left(\sqrt{\frac{\beta }{\alpha }}+\sqrt{\frac{\alpha }{\beta }}\right)-1}{S}.$$

**Figure 2:**
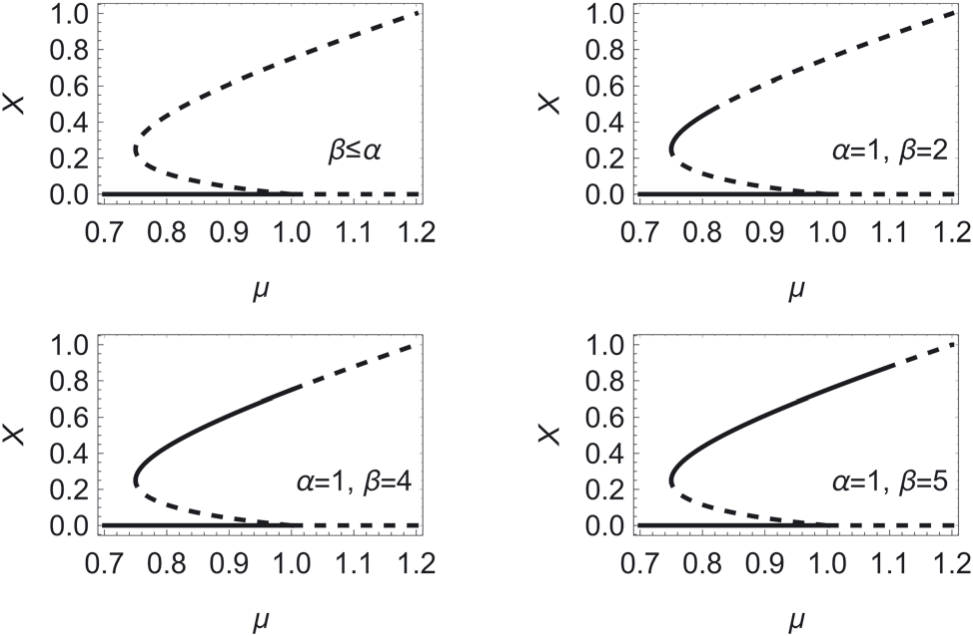
Homogeneous stationary solutions of Eq. (15) for *S* = 4. Unstable solutions are indicated with dashed lines. The laser off state is stable below the threshold *μ* = 1 and unstable above it. The negative slope branch of the nontrivial solution is always unstable. The whole upper branch of the nontrivial solution is unstable if *β* ≤ *α*, and it is stable up to the critical value *X*_*c*_ given by Eq. (19) if *β* > *α*.

If *β* < *α*, we have *X*_*c*_ < *X*_min_, which means that the critical point lies in the negative slope branch and the whole upper branch is unstable. If *α* < *β* < *Sα*, we have *μ*_min_ < *μ*_*c*_ < 1, while *μ*_*c*_ ≥ 1 if *β* ≥ *Sα*. The different possibilities are illustrated in Figure 2 for *S* = 4, *α* = 1, and various values of *β*. It is worth noting at this point that, in the case of a Mid-IR or THz QCLs, typical values of *α* and *β* as measured with different techniques range in the interval (−0.5, 2.5) [23], [24], [25], [26], [27], [28].

### 2.2 The soliton solution

Besides the homogeneous stationary solution, which corresponds to CW emission, we are interested in localized solutions or self-confined, localized structures for which the total intensity along the cavity is still stationary but the field intensity is not homogeneous. Let us introduce the total field intensity along the cavity

(20) $$I\left(\tau \right)=\overset{L/2}{\underset{-L/2}{\int }}d\eta |x\left(\eta ,\tau \right){|}^{2}.$$

For a stationary solution, we have

(21) $$\frac{dI}{d\tau }=\overset{L/2}{\underset{-L/2}{\int }}d\eta \left(\frac{\partial x}{\partial \tau }x^{*}+\frac{\partial x^{*}}{\partial \tau }x\right)=0.$$

Taking into account Eq. (15) this yields

(22) $$\overset{L/2}{\underset{-L/2}{\int }}d\eta \left(\mu -|x{|}^{2}-\frac{1}{1+S|x{|}^{2}}\right)|x{|}^{2}=0,$$

for any function *x*(*η*) such that *x*(*η*) and d*x*(*η*)/*dη* have the same value in *η* = −*L*/2 and *η* = *L*/2, as it must be.

Two trivial solutions of this equation are the laser-off state *x* = 0 and the homogeneous stationary state *μ* = |*x*|^2^ + 1/(1 + *S*|*x*|^2^). By analogy with the nonlinear Schroedinger equation, we assume that a soliton solution also exists of the form

(23) $$|x|=\sqrt{X_{s}}sech\left(\sqrt{X_{s}}\eta \right).$$

With the substitution $\eta^{\prime }=\sqrt{X_{s}}\eta$ we have

(24) $$\begin{array}{ll} \overset{\sqrt{X_{s}}L/2}{\underset{-\sqrt{X_{s}}L/2}{\int }} & d\eta^{\prime }\left[\mu \;{sech}^{2}\left(\eta^{\prime }\right)-X_{s}{sech}^{4}\left(\eta^{\prime }\right)\right.\\ & \;\left.-\frac{{sech}^{2}\left(\eta^{\prime }\right)}{1+SX_{s}{sech}^{2}\left(\eta^{\prime }\right)}\right]=0. \end{array}$$

If $L\sqrt{X_{s}}\gg 1$ , i.e., the pulse is narrow, we can extend the integration interval to (−*∞*, *∞*) and obtain an equation, which relates the peak intensity *X*_*s*_ of the pulse to the pump parameter *μ*

(25) $$\begin{array}{ll} \mu & =\overset{+\infty }{\underset{-\infty }{\int }}d\eta^{\prime }\;\left[X_{s}{sech}^{4}\left(\eta^{\prime }\right)+\frac{{sech}^{2}\left(\eta^{\prime }\right)}{1+SX_{s}{sech}^{2}\left(\eta^{\prime }\right)}\right]\\ & \;\times {\left[\overset{+\infty }{\underset{-\infty }{\int }}d\eta^{\prime }{sech}^{2}\left(\eta^{\prime }\right)\right]}^{-1}\\ & =\frac{2}{3}X_{s}+\frac{arctanh\sqrt{SX_{s}/\left(1+SX_{s}\right)}}{\sqrt{SX_{s}\left(1+SX_{s}\right)}}. \end{array}$$

The general soliton solution will have the form

(26) $$x\left(\eta ,\tau \right)=|x|\left(\eta \right){\text{e}}^{\text{i}\phi \left(\eta \right)-i\omega \tau }.$$

By inserting this expression in Eq. (15), we find

(27) $$\begin{array}{ll} -i\omega |x| & =\left(1-i\alpha \right)\left(\mu -|x{|}^{2}\right)|x|-\frac{\left(1-i\beta \right)|x|}{1+S|x{|}^{2}}\\ \; & \;+i\left(\frac{d^{2}|x|}{d\eta^{2}}+2i\frac{d|x|}{d\eta }\frac{d\phi }{d\eta }-|x|\frac{d^{2}\phi }{d\eta^{2}}\right). \end{array}$$

The condition for the real part of this equation to be equal to zero

(28) $$2\frac{d|x|}{d\eta }\frac{d\phi }{d\eta }=\left(\mu -|x{|}^{2}-\frac{1}{1+S|x{|}^{2}}\right)|x|,$$

provides an expression for the phase

(29) $$\phi =\frac{1}{2}\int_{-L/2}^{L/2}d\eta \;\frac{\mu -|x{|}^{2}-1/\left(1+S|x{|}^{2}\right)}{d|x|/d\eta }|x|.$$

**Figure 3:**
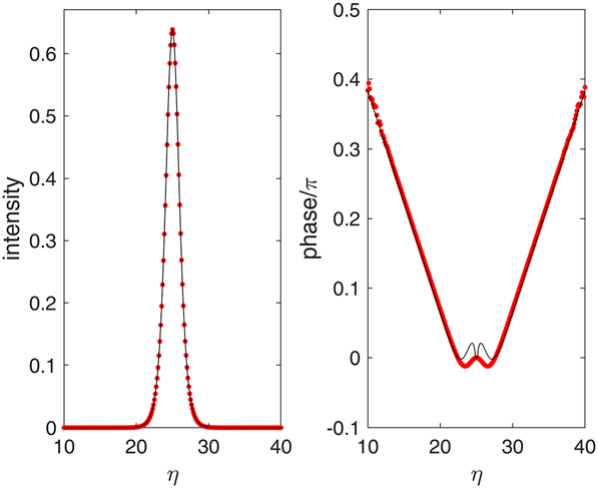
Intensity and phase profiles of the soliton solution. The black lines are the analytic approximations given by Eqs. (23) and (30), and the red symbols are the results of the numerical simulations. The parameters are *S* = 4, *α* = 2, *β* = 0, *L* = 50, and *μ* = 0.84.

With |*x*| given by Eq. (23), the substitution $\eta^{\prime }=\sqrt{X_{s}}\eta$ , and taking into account that dsech(*η*′)/*dη*′ = − tanh(*η*′) sech(*η*′), we have

(30) $$\begin{array}{ll} \phi & =-\frac{1}{2X_{s}}\int_{-\infty }^{+\infty }d\eta^{'}\;\left[\mu -X_{s}{sech}^{2}\left(\eta^{'}\right)\right.\\ & \;\left.-\frac{1}{1+SX_{s}{sech}^{2}\left(\eta^{'}\right)}\right]\frac{1}{\tanh\left(\eta^{'}\right)}\\ & =\log\left\{sech{\left(\eta^{'}\right)}^{d_{1}}\sinh{\left(\eta^{'}\right)}^{d_{2}}\times \right.\\ & \;\left.\times {\left[2SX_{s}+2\cosh^{2}\left(\eta^{'}\right)\right]}^{d_{3}}\right\}. \end{array}$$

with *d*_1_ = 1/2, *d*_2_ = [*X*_*s*_ + 1/(1 + *SX*_*s*_) − *μ*]/(4*X*_*s*_), and *d*_3_ = *S*/[4(1 + *SX*_*s*_)]. Figure 3 shows that Eqs. (23) and (30) provide very good approximations of the actual soliton solution obtained by numerical integration of Eq. (15), especially for the intensity profile. The phase shows some discrepancy around the center of the pulse, where indeed the argument of the logarithm in Eq. (30) is zero and the phase is not defined. However, we must say that for other values of the parameters, the agreement may worsen. In particular, the soliton retains its characteristic peaked profile, but it departs from the sech profile, broadening for values of *α* → 0 (see sec. III B).

To calculate the frequency of the soliton solution, we multiply the imaginary part of Eq. (27) by |*x*| and integrate

(31) $$\begin{array}{ll} \omega \int_{-L/2}^{L/2}|x{|}^{2}d\eta & =\int_{-L/2}^{L/2}\left[\alpha \left(\mu -|x{|}^{2}\right)-\frac{\beta }{1+S|x{|}^{2}})\right]|x{|}^{2}d\eta \\ & \;-\int_{-L/2}^{L/2}|x|\frac{d^{2}|x|}{d\eta^{2}}d\eta +\int_{-L/2}^{L/2}|x|\frac{d^{2}\phi }{d\eta^{2}}d\eta . \end{array}$$

Integration by parts of the last two integrals gives

(32) $$\int_{-L/2}^{L/2}|x|\frac{d^{2}|x|}{d\eta^{2}}d\eta =-\int_{-L/2}^{L/2}{\left(\frac{d|x|}{d\eta }\right)}^{2}d\eta ,$$

(33) $$\int_{-L/2}^{L/2}|x{|}^{2}\frac{d^{2}\phi }{d\eta^{2}}d\eta =-2\int_{-L/2}^{L/2}|x|\frac{d|x|}{d\eta }\frac{d\phi }{d\eta }d\eta .$$

Taking into account Eq. (28) we see that the last integral vanishes because it coincides with that of Eq. (22). With |*x*| given by Eq. (23) and the substitution $\eta^{\prime }=\sqrt{X_{s}}\eta$ , taking into account that dsech(*η*′)/*dη*′ = − tanh(*η*′)sech(*η*′) and extending the integral to (−*∞*, + *∞*) we obtain the following expression for the frequency

(34) $$\begin{array}{ll} \omega & =\overset{+\infty }{\underset{-\infty }{\int }}d\eta^{\prime }\;\left[\alpha \left(\mu -X_{s}{sech}^{2}\left(\eta^{\prime }\right)\right)-\frac{\beta }{1+SX_{s}{sech}^{2}\left(\eta^{\prime }\right)}\right.\\ & \;\left.+X_{s}\tanh^{2}\left(\eta^{\prime }\right)\right]{sech}^{2}\left(\eta^{\prime }\right)\\ & \;\times {\left[\overset{+\infty }{\underset{-\infty }{\int }}d\eta^{\prime }{sech}^{2}\left(\eta^{\prime }\right)\right]}^{-1}\\ & =\frac{X_{s}}{3}+\left(\alpha -\beta \right)\frac{arctanh\sqrt{SX_{s}/\left(1+SX_{s}\right)}}{\sqrt{SX_{s}\left(1+SX_{s}\right)}}. \end{array}$$

This integral as well those of Eqs. (25) and (30) were calculated using the symbolic calculation software *Mathematica*.

Notice that, as for the CW solution, the pulse intensity *X*_*s*_ depends only on the pump *μ* and the saturation parameter *S*, while *α* and *β* appear in the expression for the frequency *ω*.

## 3 Numerical simulations

In this section, we present the results of numerical simulations of Eq. (15) for a set of realistic QCL parameters [17], [18], [19]. We kept fixed the saturation parameter *S* = 4 (corresponding to a ratio between the saturation intensity in the laser material and that in the saturable absorber of ∼10 ) and the cavity length *L* = 50 (corresponding to a ring length of a few mm), and we adopted various values for the pair (*α*, *β*), compatible with the semiconductor materials of our interest. The temporal coordinate *τ* is instead scaled on the photon lifetime that we assume of the order of tens of ps.

We then used the pump parameter *μ* as control parameter. The types of stable solutions depend in an essential and simple way on *α* and *β*, see Section 2.1. If *α* < *β*, modulated solutions emerge beyond the critical point (*X*_*c*_, *μ*_*c*_), typically in the form of pairs of pulses, stationary, breathing, or moving. These solutions are described in the Supplementary material. Here, we will focus on the solitonic solutions, which exist for *α* > *β*.

### 3.1 Stability of the solitons

**Figure 4:**
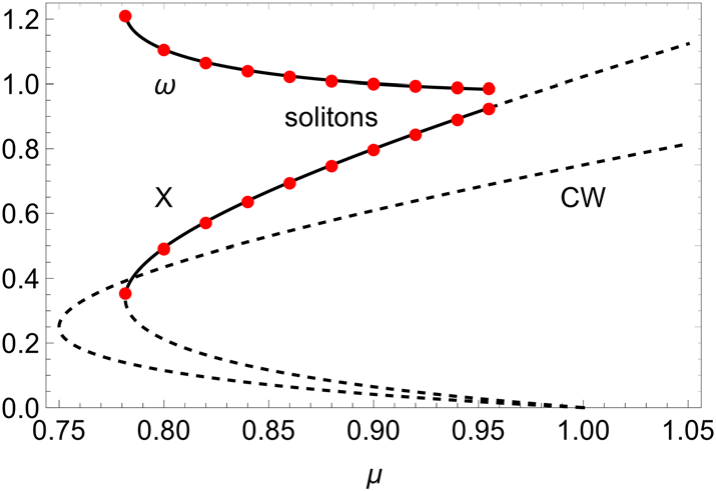
Stationary intensity and frequency of the soliton solution, as a function of the pump parameter *μ*. The black lines are the analytic curves, and the red symbols are the results of the numerical simulations for the soliton solution. The parameters are *S* = 4, *α* = 2, *β* = 0, and *L* = 50. The unstable CW solution is also shown for comparison.

The stability of the solitons was assessed by means of numerical simulations where the initial condition was the approximated solution derived above. The simulations showed that the stability depends on the parameters *α* and *β*. In Figure 4, we show the branch of stable pulses for *α* = 2 and *β* = 0. The figure shows a perfect agreement of the numerical simulations with the analytic expressions for *X* and *ω* provided by Eqs. (25) and (34), at least for that parameter choice.

**Figure 5:**
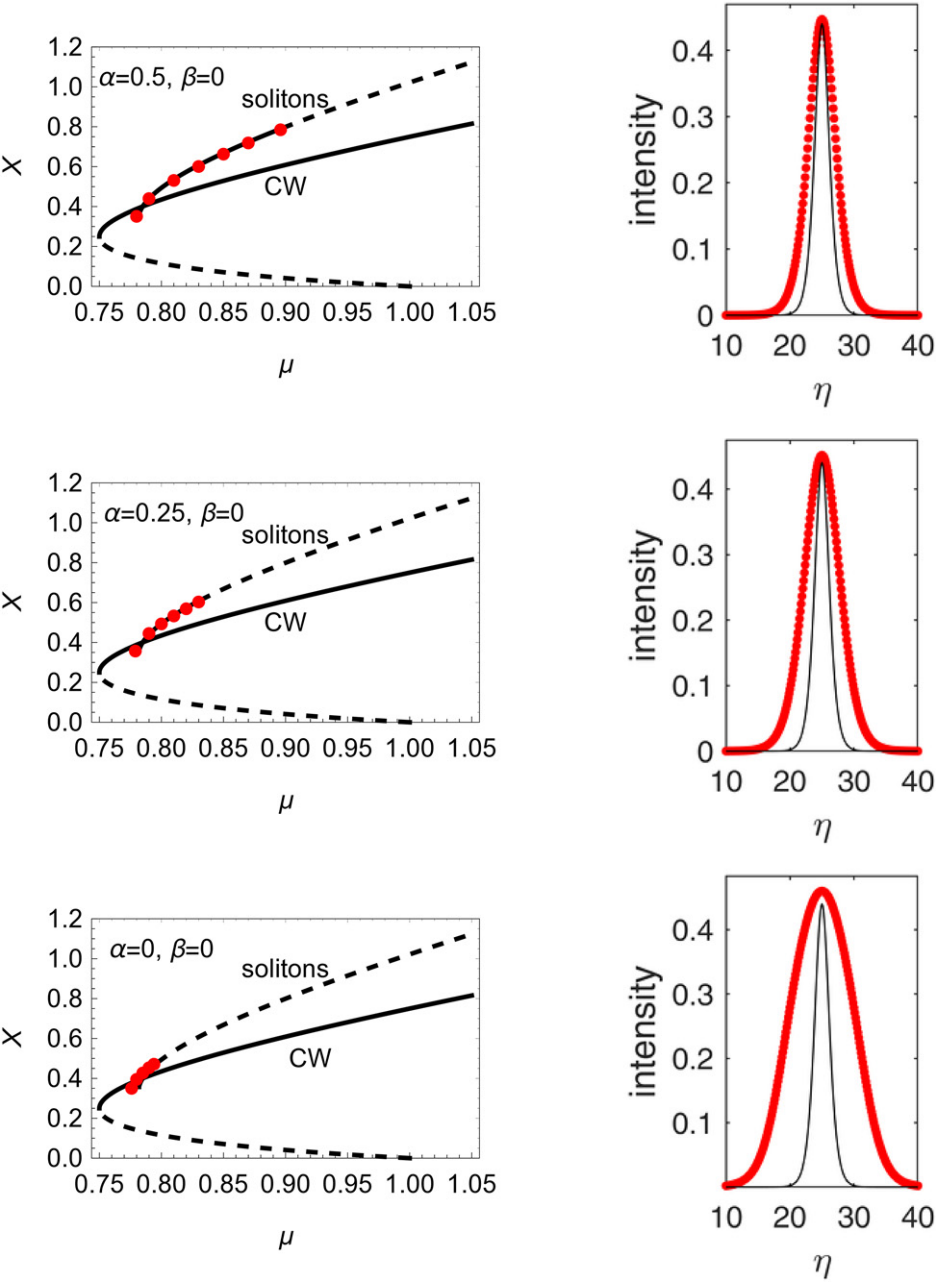
Left column: stable branch of solitons for the indicated values of *α* and *β*. The stationary intensity of the CW and sech solutions are also shown. The upper branch of the CW solution is unstable for *α* ≠ 0 and stable for *α* = 0. Right column: intensity profile of the solitons for *μ* = 0.79. The profiles calculated numerically (red symbols) are compared to the sech soliton (black lines) whose profile does not depend on *α* and *β*.

If we keep *β* = 0 and decrease *α*, we find that the soliton solution progressively departs from the sech profile calculated above, and the size of its stability domain decreases. This is illustrated in Figure 5 where in the left column we plot the branch of stable solitons for decreasing values of *α*, and in the right column the soliton profile compared to that of the sech solution. In physical units, using the scaling mentioned at the beginning of the section, the soliton width is thus of a few ps. Interestingly, there is a small branch of stable solitons even for *α* = 0, where the CW solution is modulationally stable. These solitons exist and are stable even for values of *μ* for which the sech solitons do not exist. They have a peak intensity close to that of the sech solitons, but they are much broader. These solitons, which exist even in absence of a modulational instability, were studied also by Rosanov and collaborators [6].

### 3.2 Traveling solitons

Eq. (15) is invariant under the Galilean transformation

(35) $$x\left(\eta ,\tau \right)=x^{\prime }\left(\eta^{\prime },\tau^{\prime }\right){\text{e}}^{\text{i}\left(k^{\prime }\eta -\omega^{\prime }\tau \right)},$$

with *η*′ = *η* − *vτ* and *τ*′ = *τ*. In fact,

(36) $$\frac{\partial x}{\partial \tau }=\left(\frac{\partial x^{\prime }}{\partial \tau^{\prime }}-v\frac{\partial x^{\prime }}{\partial \eta^{\prime }}-i\omega^{\prime }x^{\prime }\right){\text{e}}^{\text{i}\left(k^{\prime }\eta -\omega^{\prime }\tau \right)},$$

(37) $$\frac{\partial^{2}x}{\partial \eta^{2}}=\left(\frac{\partial^{2}x^{\prime }}{\partial {\eta^{\prime }}^{2}}+2ik^{\prime }\frac{\partial x^{\prime }}{\partial \eta^{\prime }}-{k^{\prime }}^{2}x^{\prime }\right){\text{e}}^{\text{i}\left(k^{\prime }\eta -\omega^{\prime }\tau \right)},$$

and, by inserting these expressions in Eq. (15), we see that it remains invariant with the only change *x* → *x*′, *η* → *η*′, and *τ* → *τ*′, provided *v* = 2*k*′ and *ω*′ = *k* ^′2^ = *v* ^2^/4 [6].

This means that for each stationary solution with phase *ϕ*(*η*) and frequency *ω*, there is a solution moving with velocity *v* with phase *ϕ*(*η*) + *k*′*η* and frequency *ω* − *ω*′. The velocity *v* is arbitrary.

**Figure 6:**
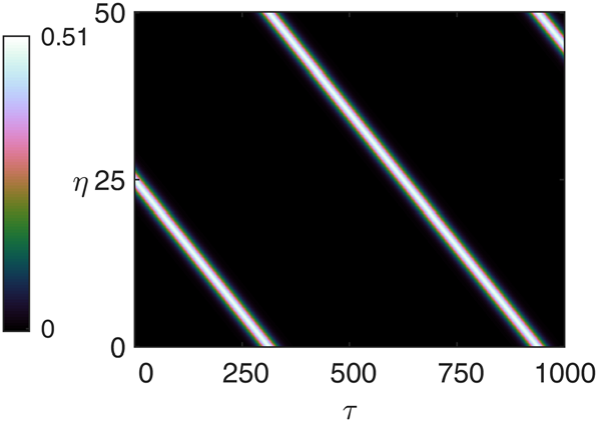
Drifting soliton for *α* = 2, *β* = 0, and *μ* = 0.8 obtained taking as initial condition the stationary soliton multiplied by the phase factor *e* ^−*ik*′*η*^, *k*′ = 0.04.

We checked this by integrating Eq. (15) taking as initial condition the stationary soliton multiplied by the phase factor *e* ^−*ik*′*η*^. The initial condition evolves into a soliton traveling at velocity *v* = 2*k*′ for any choice of *k*′, both positive and negative. Figure 6 is an example with *k*′ = 0.04. The soliton travels to the right with velocity *v* = 2*k*′ = 0.08 as it can be verified by observing that it covers one cavity length *L* = 50 in 625 time units.

**Figure 7:**
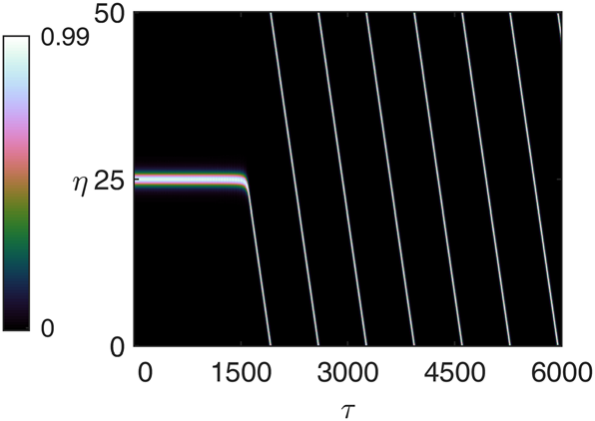
Transition from a stationary to a traveling soliton for *α* = 4, *β* = 2, and *μ* = 0.94. The initial condition is the stationary soliton for *μ* = 0.93.

The numerical simulations showed that for certain values of *α* and *β* with *α* > *β*, a spontaneous transition from stationary to traveling solitons may occur. For instance, for *α* = 4 and *β* = 2, the transition was observed around *μ* = 0.94. This is shown in Figure 7 where the initial condition is the stationary soliton found for *μ* = 0.93. After about 1,500 roundtrips, the stationary soliton starts traveling with the velocity *v* = 0.00145, which corresponds to the wavevector *k*′ = 0.000725. Repeated simulations with different initial seeds for the random number generator used to simulate noise in the system showed that indeed different wavevectors close to *k*′ can be excited. This indicates that the stationary soliton is unstable with respect to a narrow band of wavevectors.

We remark that a transition from traveling to stationary solitons in a very similar model of LSA was recently reported [29] and described in terms of a Hopf instability. In that case, however, the transition is reversed because the traveling soliton stops as the pump increases. Moreover, in [29], the velocity obeys a power law with respect to the distance from the bifurcation point, while in our simulations, the velocity remains almost unchanged as the pump increases. We thus assume a different character of the presently described instability, having a convective character, although a characterization thereof is left for a future, more detailed work.

## 4 Conclusions

We propose a new, compact, self-sustained configuration to generate self-confined pulses in the form of dissipative temporal solitons with zero background intensity in ultrafast semiconductor lasers, such as Quantum Cascade Lasers (QCLs). In particular, we refer to unidirectional ring QCLs with a fast saturable absorber that could be a reverse biased QC structure or made of, e.g., a graphene monolayer [17], [18]. In the realistic hypothesis of low saturation intensity of the absorber, we show that the system dynamics can be described by a master equation for the intracavity electric field. Contrary to previously studied models, our approach allows us to address analytically the CW (constant intensity) solution stability and to find an analytical approximation for the soliton solutions. It also allows us to run quick systematic simulations in the parameter space to guide future experiments.

We stress that the validity of the adiabatic elimination of the population variables, at the basis of our approach, has been confirmed by a systematic simulations performed using the complete model (Eqs. (1)–(3)), as reported in the Supplementary material. In particular, we show that temporal solitons with a duration of a few ps can be obtained in the complete model for typical gain and absorption recovery times of a few hundreds of fs for a cavity a few mm long.

Besides the importance of our results from a fundamental viewpoint in the field of nonlinear optics, the evidence of soliton formation in the system would certainly have a relevant impact on applications to, e.g., metrology and sensing because of the actual lack of compact and efficient emitters of mid-IR and THz short pulses.

## Supplementary Material

Supplementary Material Details

## References

[1] Lugiato L. A., Prati F., Gorodetsky M. L., Kippenberg T. J. (2018). From the Lugiato–Lefever equation to microresonator-based soliton Kerr frequency combs. *Philos. Trans. R. Soc. A*.

[2] Columbo L. (2021). Unifying frequency combs in active and passive cavities: temporal solitons in externally driven ring lasers. *Phys. Rev. Lett.*.

[3] Salomaa R., Stenholm S. (1973). Gas laser with saturable absorber. I. Single-mode characteristics. *Phys. Rev. A*.

[4] Salomaa R., Stenholm S. (1973). Gas laser with saturable absorber. II. Single-mode stability. *Phys. Rev. A*.

[5] Lugiato L. A., Mandel P., Dembinski S. T., Kossakowski A. (1978). Semiclassical and quantum theories of bistability in lasers containing saturable absorbers. *Phys. Rev. A*.

[6] Rosanov N. N. (2002). *Spatial Hysteresis and Optical Patterns*.

[7] Fedorov S. V., Vladimirov A. G., Khodova G. V., Rosanov N. N. (2000). Effect of frequency detunings and finite relaxation rates on laser localized structures. *Phys. Rev. E*.

[8] Haus H. A. (2000). Mode-locking of lasers. *IEEE J. Sel. Top. Quantum Electron.*.

[9] Soto-Crespo J. M., Akhmediev N. N., Afanasjev V. V. (1996). Stability of the pulselike solutions of the quintic complex Ginzburg-Landau equation. *J. Opt. Soc. Am. B*.

[10] Grelu P., Akhmediev N. (2012). Dissipative solitons for mode-locked lasers. *Nat. Photonics*.

[11] Lugiato L. A., Lefever R. (1987). Spatial dissipative structures in passive optical systems. *Phys. Rev. Lett.*.

[12] Lugiato L. A., Oldano C., Narducci L. M. (1988). Cooperative frequency locking and stationary spatial structures in lasers. *J. Opt. Soc. Am. B*.

[13] Bache M. (2005). Cavity soliton laser based on vcsel with saturable absorber. *Appl. Phys. B*.

[14] Marconi M., Javaloyes J., Balle S., Giudici M. (2014). How lasing localized structures evolve out of passive mode locking. *Phys. Rev. Lett.*.

[15] Faist J., Capasso F., Sivco D. L., Sirtori C., Hutchinson A. L., Cho A. Y. (1994). Quantum cascade laser. *Science*.

[16] Piccardo M., Capasso F. (2022). Laser frequency combs with fast gain recovery: Physics and applications. *Laser Photon. Rev.*.

[17] Riccardi E. (2023). Short pulse generation from a graphene-coupled passively mode-locked terahertz laser. *Nat. Photonics*.

[18] Seitner L., Popp J., Haider M., Dhillon S. S., Vitiello M. S., Jirauschek C. (2024). Theoretical model of passive mode-locking in terahertz quantum cascade lasers with distributed saturable absorbers. *Nanophotonics*.

[19] Faist J. (2013). *Quantum Cascade Lasers*.

[20] Micheletti P. (2023). Terahertz optical solitons from dispersion-compensated antenna-coupled planarized ring quantum cascade lasers. *Sci. Adv.*.

[21] Henry C. H. (1982). Theory of the linewidth of semiconductor lasers. *IEEE J. Quantum Electron.*.

[22] Prati F., Columbo L. (2007). Long-wavelength instability in broad-area semiconductor lasers. *Phys. Rev. A*.

[23] Aellen T. (2006). Direct measurement of the linewidth enhancement factor by optical heterodyning of an amplitude-modulated quantum cascade laser. *Appl. Phys. Lett.*.

[24] von Staden J., Gensty T., Elsaesser W., Giuliani G., Mann C. (2006). Measurements of the a factor of a distributed-feedback quantum cascade laser by an optical feedback self-mixing technique. *Opt Lett.*.

[25] Hangauer A., Wysocki G. (2015). Gain compression and linewidth enhancement factor in mid-ir quantum cascade lasers. *IEEE J. Sel. Top. Quantum Electron.*.

[26] Jumpertz L. (2016). Measurements of the linewidth enhancement factor of mid-infrared quantum cascade lasers by different optical feedback techniques. *AIP Adv.*.

[27] Piccardo M. (2020). Frequency combs induced by phase turbulence. *Nature*.

[28] Opačak N. (2021). Spectrally resolved linewidth enhancement factor of a semiconductor frequency comb. *Optica*.

[29] Humire F. R., Alfaro-Bittner K., Clerc M. G., Rojas R. G. (2024). Transition from traveling to motionless pulses in semiconductor lasers with saturable absorber. *Phys. D*.

