# Supplementary material for: Solitons in ultrafast semiconductor lasers with saturable absorber
Source: Nanophotonics. 2025 Aug 28;14(21):3459–67. doi: 10.1515/nanoph-2025-0057 (PMC12552883; doi:10.1515/nanoph-2025-0057)
Supplement: Supplementary file 1 — Supplementary Material Details [file j_nanoph-2025-0057_suppl_001.pdf]

Supplementary Material

Modulational instability for $\alpha < \beta$

With the choice of the parameters $\alpha = 1$ and $\beta = 2$ the plot of the CW solution with its stable and unstable branches is the one at the top right corner of Fig. 2. We reproduce it in Fig. 8 where we also indicate with the labels (a), (b), (c), and (d) the minimum and maximum intensity of some interesting solutions on which we will focus.

According to Eq. (19), for $S = 4$, $\alpha = 1$, and $\beta = 2$, the upper branch is unstable above $\mu_c \simeq 0.81$.

At $\mu = 0.83$ we observe a periodic solution formed by two identical pulses which are strong modulations of the CW solution, although their minimum intensity remains far from zero (Fig. 9(a)).

By increasing the pump μ, at $\mu = 0.845$ the previous periodic solution displays an instability which occurs on very long time scales, on the order of some thousands of roundtrips (Fig. 9(b)). As shown by Fig. 10 upper left frame, the instability consists in the merging of the two pulses into a single pulse at an intermediate position, followed immediately later by the appearance of a second pulse at a distance of half a cavity from the other pulse. The net result is a shift of the two pulses by one fourth of the cavity length. Then, in a periodic manner, the two pulses shift in the other position. As we will see, this process involves the odd modes while in the 2-pulse solution of Fig. 9(a) only the even modes are present.

At $\mu = 0.88$ we observe again a 2-pulse solution, but now the two pulses have slightly different heights and the higher one has two small satellites at its sides (Fig. 9(c)). Finally, at $\mu = 0.915$ the previous 2-pulse solution starts drifting (Fig. 9(d)). For higher values of μ only turbulent dynamics are found.

In Fig. 10 we analyse in detail the switch of the 2-pulse solution described previously. The spatio-temporal plot in the top left corner is a zoom around the transition which shows the two phases of the process: first two pulses merge, then a second pulse is generated. The plot in the top right corner shows that the transition is accompanied by a very small burst in the maximum intensity which occurs when the two pulses merge. In the bottom part of the figure we see that before and after the switch only even modes are active, and odd modes are excited just for a short time interval during the switch. The phases of the modes are constant before and after the switch, but during the switch

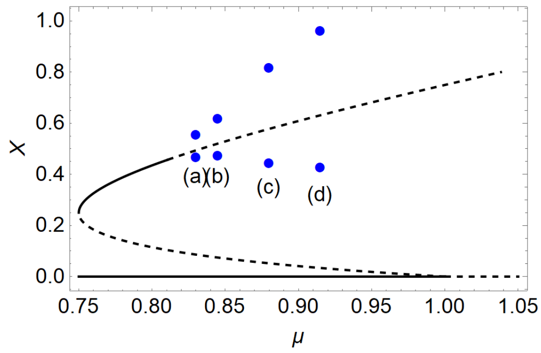

Fig. 8: Stationary intensity of the CW solution, as a function of the pump parameter μ. The upper branch is stable up to $\mu_c = 0.81$. The symbols indicate the minimum and maximum intensity of the modulated solutions for (a) $\mu = 0.83$, (b) $\mu = 0.845$, (c) $\mu = 0.88$, and (d) $\mu = 0.915$. The parameters are $S = 4$, $\alpha = 1$, $\beta = 2$, $L = 50$.

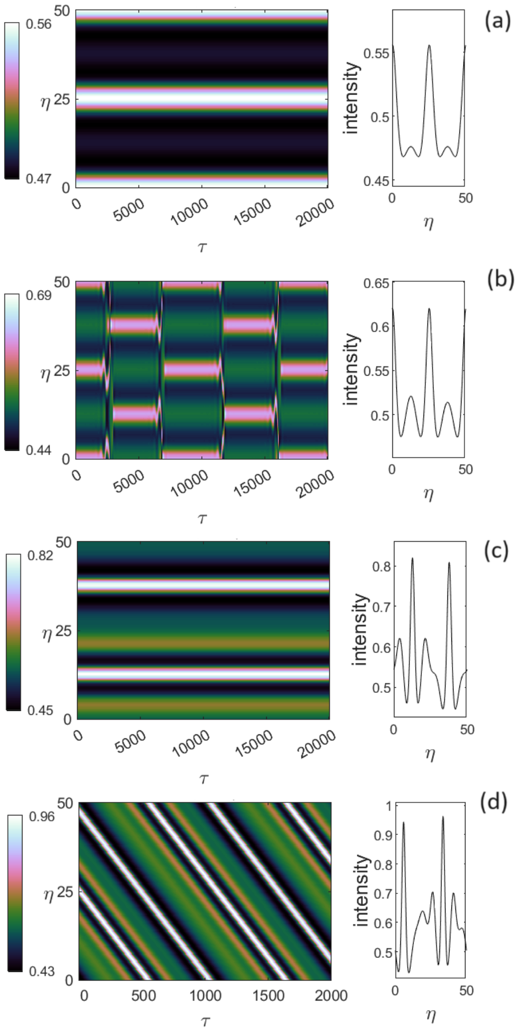

Fig. 9: Dynamical regimes corresponding to the labels (a,b,c,d) of the previous figure. In each subplot we show the spatiotemporal evolution of the field intensity (left) and the intensity profile (right) at the final time.

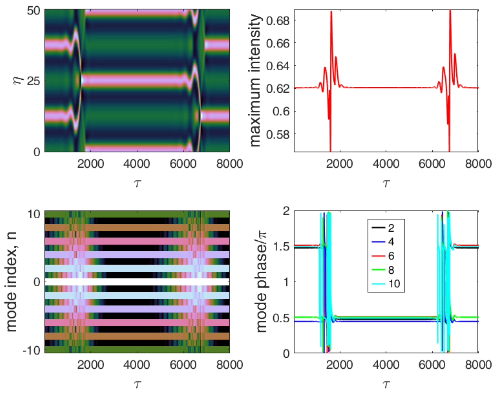

Fig. 10: Analysis of the shift of the 2-pulse solution which characterises the dynamical regime (b) of the previous figure. In correspondence with every shift, odd modes are temporarily excited, the total intensity displays a small burst and the phases of the modes whose index is an odd multiple of 2 (modes 2, 6, and 10 in the figure) experience a jump of π.

they are scrambled and the modes whose index is an odd multiple of 2 (modes 2, 6, and 10 in the figure) emerge from the switch shifted by π, while the modes whose index is a multiple of 4 return to their previous phase. This agrees with the shift by one fourth of the cavity length which occurs after the switch because, if we expand the electric field in terms of the longitudinal modes $\exp(i2\pi n\eta/L)$ with complex amplitudes $x_n(\tau)$, we have

$$x(\tau,\eta \pm L/4) = \sum x_n(\tau) e^{i2\pi n(\eta \pm L/4)/L} \qquad (38)$$
$$= \sum x_n(\tau) e^{\pm in\pi/2} e^{i2\pi n\eta/L} = \sum x'_n(\tau) e^{i2\pi n\eta/L},$$

with

$$x'_n(\tau) = x_n(\tau) e^{\pm in\pi/2}, \qquad (39)$$

thus $x'_n(\tau) = x_n(\tau)$ if $n = 4m$ and $x'_n(\tau) = -x_n(\tau)$ if $n = (2m+1)2$, with m integer.

Comparison between the complete model and the reduced one

In deriving from the full set of Eqs. (1)-(3) the reduced model whose final form is Eq. (15) we made two assumptions: i) the material variables D and $\bar{D}$ evolve on a temporal scale much faster than the field envelope F, and ii) the laser is sufficiently close to threshold. Assumption i) allows us to adiabatically eliminate the equations for D and $\bar{D}$, assumption ii) implies that the scaled field intensity $|F|^2$ is a quantity much smaller than 1, and this allows us to make a cubic approximation in the gain, while the full nonlinearity is kept in the absorption since we also assume that the saturation parameter s is much larger than 1 and therefore the product $s|F|^2$ is of order 1.

The validity of the above assumption was checked by numerical integration of the set of equations (1)-(3) with $\bar{A} = 0.4$ and $s = 10$. As shown in Fig. 1, for this choice of the parameters (which leads to $S = 4$ in the reduced model) the stationary intensity in the full and reduced model are very close one to the other. In the region of interest, below the threshold $A_{th} = 1 + \bar{A} = 1.4$, the scaled intensity takes values around 0.2, which are consistent with the cubic approximation for the gain.

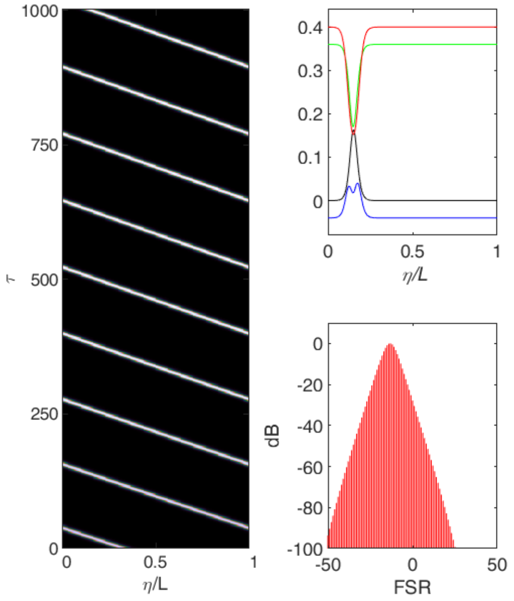

Fig. 11: Soliton solution according to the full set of equations (1-3). The left panel shows the motion of the soliton in the (η,τ) plane, which is not a vertical line because the full model is written in the laboratory frame, not in the comoving frame as the reduced model. The upper right panel shows the profile of the field intensity I (black), the gain minus the linear losses $D-1$ (green), the nonlinear losses $\bar{D}$ (red), and the net gain $D-\bar{D}-1$ (blue). The lower left panel shows the spectrum associated with the soliton, which is centered around mode of index -14.

A relevant point is how fast the material variables must be to justify their adiabatic elimination, considering that in presence of pulses the relevant time scale for the field F is not given by the cavity damping rate κ but rather by the duration of the pulse. We scaled the time variable to the cavity roundtrip time $\mathcal{L}/\tilde{c}$, where $\mathcal{L}$ is the cavity length in physical units (different from L) so that if we name τ and $\bar{\tau}$ the recovery times of

the amplifier and of the absorber, respectively, we have $\gamma = \mathcal{L}/\tilde{c}\tau$ and $\bar{\gamma} = \mathcal{L}/\tilde{c}\tilde{\tau}$ and the question is how large must γ and $\bar{\gamma}$ be. We set $\alpha = 2$ and $\beta = 0$ to check that the soliton solution in Fig. 3 persists in the full set of equations. We also set $d = 1.6 \times 10^{-4}$, which corresponds to the cavity length $L = 50$ in dimensionless units. We fixed the pump parameter $A = 1.36$, below the threshold $A = 1 + \bar{A} = 1.4$, and made several simulations starting with large values of the parameters γ and $\bar{\gamma}$, for which the adiabatic elimination of D and $\bar{D}$ is certainly justified, and reducing them until the soliton solution disappeared. We found stable solitons for $\gamma = \bar{\gamma} = 400$, which become unstable for $\gamma = \bar{\gamma} = 300$. Hence, the soliton solution of the reduced model survives in the full model if $\gamma = \mathcal{L}/\tilde{c}\tau = 400$. Since $\tilde{c} \sim 10^8$ m/s this means $\tau = (\mathcal{L}/4) \times 10^{-10}$ s/m or $\tau(\text{ps}) = 0.025 \times \mathcal{L}(\text{mm})$. Therefore, the soliton may exist in cavities some mm long if the recovery times of the materials are on the order of a few hundreds of fs. Figure 11 shows the soliton solution obtained by integrating the full set of equations with the above mentioned parameters. The pulse duration is about one tenth the roundtrip time, which means a few ps for a cavity of a few mm long as for the reduced model integrated in section 3. The fast recovery times of the material variables allow them to follow almost instantaneously the intensity variations associated with the pulse. The window of positive net gain (blue line in the upper right panel of the figure) presents two maxima. We remark that in order to stabilize the soliton solution in the complete model we added a small real part to the coefficient to the coefficient of the second order derivative in Eq. (1), equal to $0.02d$. This turns out to be not necessary in the reduced model Eq. (15). This term provides a gain filter mechanism which fixes the maximum gain frequency. In particular, this sets the max of the soliton spectrum around mode -14.
